# Safe Treatment of an Extensive-Stage Small-Cell Lung Cancer With Tarlatamab in an Orthotopic Heart Transplantation Patient: A Case Report

**DOI:** 10.7759/cureus.92622

**Published:** 2025-09-18

**Authors:** Amr Ismail, Tingting Zhang, Aaron Vickers, Drexell H Boggs, Aakash Desai, Yanis Boumber

**Affiliations:** 1 Department of Medicine, Division of Hematology and Oncology, University of Alabama at Birmingham School of Medicine, Birmingham, USA; 2 Department of Oncology, Shengli Oilfield Central Hospital, Dongying, CHN; 3 Department of Radiation Oncology, University of Alabama at Birmingham School of Medicine, Birmingham, USA

**Keywords:** bispecific antibody, case report, heart transplant, small-cell lung cancer, tarlatamab

## Abstract

Graft rejection is the major challenge that solid organ transplant recipients face. As a consequence, lung cancer patients of this population have been consistently excluded from clinical trials involving the use of immunotherapy agents due to the increased risk of graft rejection. Tarlatamab is a novel bi-specific T-cell recruiter monoclonal antibody targeting delta-like ligand 3 (DLL3) on cancer cells and CD3 on T-cells; hence, it is theoretically much safer than other checkpoint inhibitor immunotherapy agents that boost T-cells non-specifically. Tarlatamab received Food and Drug Administration (FDA) approval in 2024 for the treatment of adults with extensive-stage small-cell lung cancer (ES-SCLC) that has progressed on or after platinum-based chemotherapy. Here, we report safe treatment of an extensive-stage small-cell lung cancer patient with an orthotopic heart transplant with tarlatamab. The patient received four doses between April and June 2025 without evidence of graft rejection or dysfunction. As of August 2025, the patient continues treatment without any adverse events.

## Introduction

Solid organ transplant recipients (SOTRs) represent an interesting population in the context of oncology. Cancer is the second most common cause of death in this group [1], largely attributed to the chronic immunosuppression required to mitigate graft rejection [2,3]. For the same reason, SOTRs are typically excluded from immunotherapy trials. Immune checkpoint inhibitors (ICIs) in particular carry a substantial risk of allograft rejection, reported in one multicenter series of 39 patients at 41% overall, with organ-specific rates of 48% in renal, 36% in hepatic, and 20% in cardiac transplant recipients [4]. A systematic review of published cases found rejection rates of 60% overall, including 73% with nivolumab and 100% with pembrolizumab, while no rejections occurred with ipilimumab monotherapy [5]. A pharmacovigilance analysis of 96 reported cases further showed that most events occurred in kidney and liver recipients, were predominantly associated with anti-PD-1/PD-L1 therapy (93%), and frequently led to graft loss and death [6].

Tarlatamab, a delta-like ligand 3 (DLL3) bispecific T-cell engager (BiTE), has recently demonstrated promising activity in relapsed small-cell lung cancer. In the pivotal phase 3 trial, cytokine release syndrome (CRS) occurred in 56% of patients, nearly all grade 1-2 (42% and 13%, respectively), with only 1% experiencing grade 3 and no grade 4-5 cases. Neurologic adverse events were also reported in 56% of patients, including immune effector cell-associated neurotoxicity syndrome (ICANS) in 6%, which was generally low grade [7].

Here, we report the safe use of tarlatamab, a DLL3 bispecific T-cell engager (BiTE), in an orthotopic heart transplantation patient on chronic immunosuppression.

## Case presentation

A 69-year-old male with 25-pack-year smoking history, quit in 2019, and 2020 orthotopic heart transplant for advanced heart failure with reduced ejection fraction (EF), on tacrolimus 0.5 mg twice daily and 5 mg prednisone daily. His past medical history includes chronic kidney disease, hypertension, and hypothyroidism.

**Figure 1:**
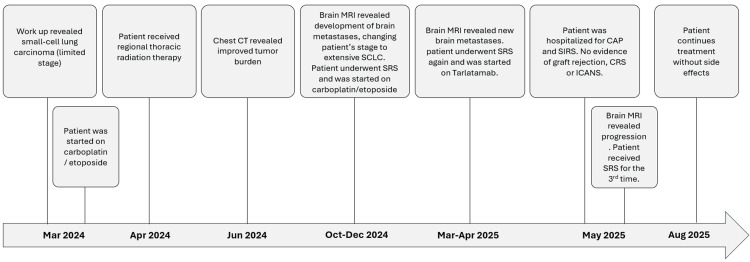
Summary of clinical course. CT: computed tomography; MRI: magnetic resonance imaging; SCLC: small-cell lung cancer; SRS: stereotactic radiosurgery; CAP: community-acquired pneumonia; SIRS: systemic inflammatory response syndrome; CRS: cytokine release syndrome; ICANS: immune effector cell-associated neurotoxicity syndrome.

The course of treatment is summarized in Figure 1. The patient presented to the thoracic medical oncology clinic with two weeks of productive cough, fatigue, and dyspnea. He had Eastern Cooperative Oncology Group performance status of 0. Chest, abdomen, and pelvis computed tomography (CT) without contrast revealed a 3x2.5 cm mass in the right upper lobe (RUL) with hilar and mediastinal lymphadenopathy and no metastasis. Biopsies 03/2024 revealed small-cell lung cancer (SCLC).

**Figure 2:**
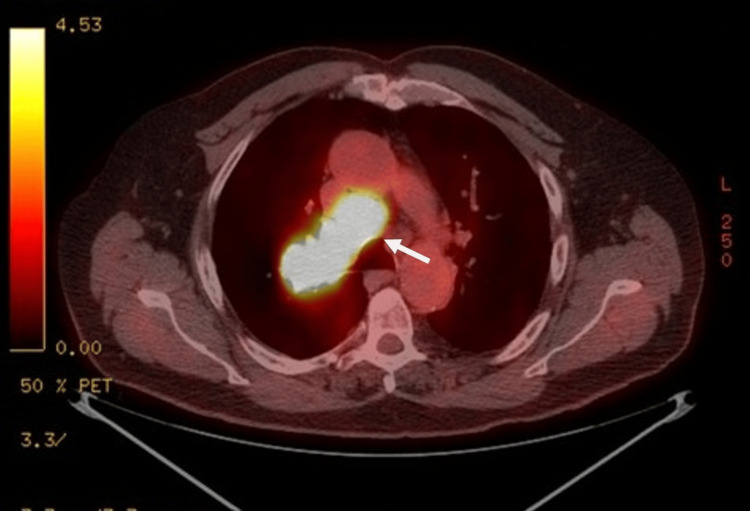
Axial PET/CT demonstrating hypermetabolic right hilar mass. An axial PET/CT image shows intense uptake (white arrow) in the right hilar region. PET-CT: positron emission tomography-computed tomography.

Positron emission tomography revealed hypermetabolic RUL mass extending to the hilum and hypermetabolic mediastinal adenopathy (Figure 2). Brain magnetic resonance imaging (MRI) with contrast revealed no metastases, consistent with limited-stage SCLC.

The patient started intravenous carboplatin (AUC 5) and etoposide (100 mg/m^2^) on 03/2024, with a total of four 21-day cycles planned, infusions on days one to three of each cycle (03/2024-05/2024). Twenty days after starting chemotherapy, chest radiotherapy was delivered over six weeks. In June 2024, CT scans revealed improvement of the RUL mass and the hilar/mediastinal lymphadenopathy.

**Figure 3:**
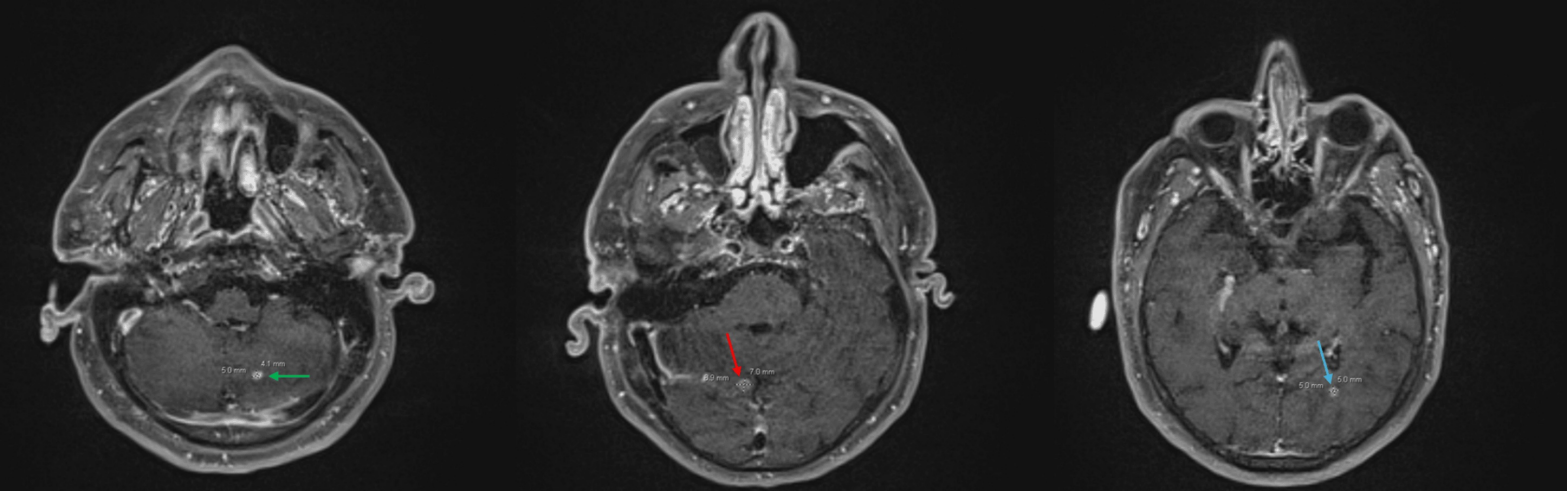
Axial brain MRI showing multiple brain metastases. Post-contrast images of axial brain MRI showing enhancing lesions in the left cerebellar hemisphere (green arrow), right posterior lateral splenic corpus callosum (red arrow), and left occipital lobe (blue arrow).

Surveillance brain MRI on 10/2024 showed multiple metastatic lesions, indicating progression to extensive-stage SCLC (Figure 3). He underwent stereotactic radiosurgery (SRS) on 11/2024. A second course of carboplatin/etoposide, x4 cycles, was administered using the same doses and schedules as his initial treatment, 11/2024-12/2024, with the addition of pegfilgrastim. PD-L1-directed immunotherapy was not used due to unacceptable risks of toxicities or death in SOTRs [4]. Unfortunately, a brain MRI three months later revealed new lesions, for which he underwent SRS for the second time on 4/2025.

The patient started on second-line tarlatamab in 04/2025, given its known intracranial efficacy, with reported intracranial responses (≥30% tumor shrinkage) observed in 62.5% of patients with measurable brain metastases [8]. He was admitted twice for observation per guidelines post 1 mg, day one and 10 mg on day eight [9]. Subsequent 10 mg doses (C1D15 onward) were given outpatient with same-day monitoring every two weeks. The patient tolerated tarlatamab well with no cytokine release syndrome (CRS), immune effector cell-associated neurotoxicity syndrome (ICANS), or graft rejection.

Prior to cycle two of tarlatamab 5/2025, he was hospitalized due to fever, cough, and dyspnea. He was diagnosed with acute hypoxic respiratory failure, multifocal community-acquired pneumonia, and systemic inflammatory response syndrome (SIRS). Importantly, no CRS, graft rejection, or ICANS was observed. Polymerase chain reaction (PCR) testing was positive for rhinovirus/enterovirus. He received cefepime and subsequently levofloxacin with marked improvement upon discharge. Comprehensive cardiac assessment, including echocardiography and biomarker-based rejection surveillance, showed no signs of graft dysfunction: echocardiogram 4/2025 before tarlatamab initiation showed ejection fraction (EF) of 65-70% and B-type natriuretic peptide (BNP) of 74 pg/ml. Upon the patient's admission, echocardiogram showed EF of 60-65% and BNP of 54 pg/ml. 

In May 2025, restaging CT scans showed no disease. However, brain MRI showed progressive lesions, and the patient received SRS for the third time on 5/2025. He continued cycle two tarlatamab (10 mg every two weeks) in June-July 2025 with no side effects. These findings suggest a lack of intracranial response, with brain-only progression and no extracranial disease. Continuation of tarlatamab is planned after repeating brain MRI and CTs in September 2025. As of August 2025, the patient continues treatment without any adverse events.

## Discussion

Immunotherapy, especially non-specific T-cell boosters/checkpoint inhibitors, remains controversial in SOTRs due to graft rejection risk [4]. This case represents, to our knowledge, the first reported use of tarlatamab in a SOTR, specifically SCLC patient with a history of orthotopic heart transplantation. He started tarlatamab therapy on 04/2025 and received a total of four doses without evidence of graft rejection or dysfunction. This heart transplant recipient was closely monitored by the transplant cardiology team in both outpatient and inpatient settings. He also underwent inpatient follow-up with the oncology and hematology teams experienced in administering bispecific T-cell engagers (BiTEs) and chimeric antigen receptor T-cell (CAR-T) therapies, following standard cancer-center protocols. After two initial weekly doses, laboratory testing and clinical evaluations were performed every two weeks, consistent with other patients receiving tarlatamab, with the exception of additional cardiology assessments, including BNP measurements and echocardiograms at frequent intervals.

Recent publications challenge the longstanding exclusion of solid organ transplant recipients from trials (Table 1). Notably, case reports support cautious use of T-cell-engaging immunotherapies in SOTRs. Unlike checkpoint inhibitors with broad T-cell activation and high rejection risk [4], CAR‑T and bispecifics demonstrate targeted activity and likely acceptable safety in SOTRs. Case series of 17 SOTR patients (kidney (n = 12), liver (n = 2), heart (n = 2), and pancreas following kidney transplant (n = 1)) treated with CD19-CAR‑T (axicabtagene, lisocabtagene, tisagenlecleucel) for post‑transplant lymphoproliferative disorder showed an 82% overall response rate and 59% complete remissions, with graft rejection seen in 23.5% of cases, all of them were kidney transplant recipients. Among the overall and axicabtagene cohorts, grade ≥3 ICANS occurred in 29.4% (5/17) and 36.4% (4/11). No cases of graft failure or rejection-related mortality occurred [10].

**Table 1:** Selected reports of CAR-T and bispecific agents use in transplant recipients. CAR-T: chimeric antigen receptor T-cell; PTLD: post-transplant lymphoproliferative disorder; DLBCL: diffuse large B-cell lymphoma; ORR: overall response rate; CR: complete remission; BiTE: bi-specific T-cell engager; B-ALL: B-cell acute lymphocytic leukemia; HSCT: hematopoietic stem cell transplantation; GVHD: graft-versus-host disease; CRS: cytokine release syndrome; EBV: Epstein-Barr virus; DLL3: delta-like ligand 3; SCLC: small-cell lung cancer; ICANS: immune effector cell-associated neurotoxicity syndrome.

Study, year	Agent	Indication	Transplant type	Outcome	Graft rejection
Portuguese et al. 2023 [10]	CD19-CAR-T (axi-cel, liso-cel, tisa-cel)	PTLD (DLBCL/Burkitt)	Kidney, liver, heart	ORR: 82%, CR: 59%; manageable toxicity	4/17 cases
Stein et al. 2019 [11]	Blinatumomab (CD19×CD3 BiTE)	B-ALL post-HSCT	Allogeneic HSCT	CR: ~45%; minimal GVHD; tolerable CRS/neurotoxicity	Minimal GVHD
Chen et al. 2023 [12]	Blinatumomab (CD19×CD3 BiTE)	EBV-related PTLD	Allogeneic HSCT	CR achieved; no graft rejection	No
This report	Tarlatamab (DLL3×CD3)	SCLC	Heart (orthotopic)	Ongoing treatment; no CRS, ICANS, or graft rejection	No

Blinatumomab (CD19×CD3 bispecific antibody) has also been used after allogeneic hematopoietic stem cell transplantation (HSCT) in patients with relapsed/refractory B-ALL, achieving remission in 45% of 64 patients with minimal grade 3 graft-versus-host disease (GVHD) seen in seven patients (11%), none of which required discontinuation of the drug or hospitalization, therefore manageable toxicity [11]. A recent report documented successful treatment of an EBV-related PTLD case in an allogeneic HSCT recipient with blinatumomab, achieving complete remission without graft rejection [12].

Our patient did not respond to tarlatamab; however, he had no major toxicities except pneumonia, which was treated with antibiotics. This demonstrates the potential for personalized immunotherapy in transplant recipients with malignancy. It opens the door for further exploration of BiTEs like tarlatamab in SOTR patients using trials or registries. To contextualize our report, we conducted a literature search in PubMed and Embase, supplemented by Google Scholar (last accessed August 2025), using combinations of the terms “tarlatamab,” “DLL3,” “bispecific T-cell engager,” “small-cell lung cancer,” “immunotherapy,” “solid organ transplant,” “heart transplant,” “graft rejection,” “cytokine release syndrome,” “immune effector cell-associated neurotoxicity syndrome.” This search did not identify any prior cases describing the use of tarlatamab in solid organ transplant recipients. To our knowledge, this represents the first reported use of tarlatamab in a heart transplant recipient with recurrent extensive-stage small-cell lung cancer. The patient tolerated it well, suggesting that tumor-targeted bispecific agents could be safe in SOTRs. More studies of DLL3-targeting and other bispecifics in patients with organ transplants receiving immunosuppression are needed.

## Conclusions

This case highlights the first reported use of tarlatamab in a heart transplant recipient with ES-SCLC. Despite chronic immunosuppression, the patient tolerated the drug well, with no evidence of graft rejection or major immune-related toxicity. Although intracranial progression occurred, extracranial disease remained controlled. These findings support further investigation of bispecific agents in solid organ transplant recipients through prospective studies or registries.

## References

[1] Acuna SA, Fernandes KA, Daly C, Hicks LK, Sutradhar R, Kim SJ, Baxter NN (2016). Cancer mortality among recipients of solid-organ transplantation in Ontario, Canada. JAMA Oncol.

[2] Papaconstantinou HT, Sklow B, Hanaway MJ (2004). Characteristics and survival patterns of solid organ transplant patients developing de novo colon and rectal cancer. Dis Colon Rectum.

[3] Taylor AL, Marcus R, Bradley JA (2005). Post-transplant lymphoproliferative disorders (PTLD) after solid organ transplantation. Crit Rev Oncol Hematol.

[4] Abdel-Wahab N, Safa H, Abudayyeh A (2019). Checkpoint inhibitor therapy for cancer in solid organ transplantation recipients: an institutional experience and a systematic review of the literature. J Immunother Cancer.

[5] Aguirre LE, Guzman ME, Lopes G, Hurley J (2019). Immune checkpoint inhibitors and the risk of allograft rejection: a comprehensive analysis on an emerging issue. Oncologist.

[6] Nguyen LS, Ortuno S, Lebrun-Vignes B, Johnson DB, Moslehi JJ, Hertig A, Salem JE (2021). Transplant rejections associated with immune checkpoint inhibitors: a pharmacovigilance study and systematic literature review. Eur J Cancer.

[7] Mountzios G, Sun L, Cho BC (2025). Tarlatamab in small-cell lung cancer after platinum-based chemotherapy. N Engl J Med.

[8] Dowlati A, Hummel HD, Champiat S (2024). Sustained clinical benefit and intracranial activity of tarlatamab in previously treated small cell lung cancer: DeLLphi-300 trial update. J Clin Oncol.

[9] (2025). Amgen Inc. Imdelltra (tarlatamab-dlle) prescribing information. https://www.accessdata.fda.gov/drugsatfda_docs/label/2024/761344s000lbl.pdf.

[10] Portuguese AJ, Gauthier J, Tykodi SS, Hall ET, Hirayama AV, Yeung CC, Blosser CD (2023). CD19 CAR-T therapy in solid organ transplant recipients: case report and systematic review. Bone Marrow Transplant.

[11] Stein AS, Kantarjian H, Gökbuget N (2019). Blinatumomab for acute lymphoblastic leukemia relapse after allogeneic hematopoietic stem cell transplantation. Biol Blood Marrow Transplant.

[12] Chen S, An L, Han J (2023). Successful Blinatumomab treatment in an allogeneic hematopoietic stem cell transplant recipient with EBV-related post-transplant lymphoproliferative disorder: a case report and literature review. Transpl Immunol.

